# Carbon metabolism and biogeography of candidate phylum “*Candidatus* Bipolaricaulota” in geothermal environments of Biga Peninsula, Turkey

**DOI:** 10.3389/fmicb.2023.1063139

**Published:** 2023-02-22

**Authors:** Ömer K. Coskun, Gonzalo V. Gomez-Saez, Murat Beren, Dogacan Ozcan, Hakan Hosgormez, Florian Einsiedl, William D. Orsi

**Affiliations:** ^1^Department of Earth and Environmental Sciences, Paleontology and Geobiology, Ludwig-Maximilians-Universität München, Munich, Germany; ^2^GeoBio-Center^LMU^, Ludwig-Maximilians-Universität München, Munich, Germany; ^3^Department of Geological Engineering, Istanbul University-Cerrahpasa, Istanbul, Türkiye; ^4^Chair of Hydrogeology, TUM School of Engineering and Design, Technical University of Munich, Munich, Germany

**Keywords:** “*Candidatus* Bipolaricaulota”, deep biosphere, hydrothermal, carbon fixation, pangenomics, phylogenomics

## Abstract

Terrestrial hydrothermal springs and aquifers are excellent sites to study microbial biogeography because of their high physicochemical heterogeneity across relatively limited geographic regions. In this study, we performed 16S rRNA gene sequencing and metagenomic analyses of the microbial diversity of 11 different geothermal aquifers and springs across the tectonically active Biga Peninsula (Turkey). Across geothermal settings ranging in temperature from 43 to 79°C, one of the most highly represented groups in both 16S rRNA gene and metagenomic datasets was affiliated with the uncultivated phylum “*Candidatus* Bipolaricaulota” (former “*Ca.* Acetothermia” and OP1 division). The highest relative abundance of “*Ca.* Bipolaricaulota” was observed in a 68°C geothermal brine sediment, where it dominated the microbial community, representing 91% of all detectable 16S rRNA genes. Correlation analysis of “*Ca.* Bipolaricaulota” operational taxonomic units (OTUs) with physicochemical parameters indicated that salinity was the strongest environmental factor measured associated with the distribution of this novel group in geothermal fluids. Correspondingly, analysis of 23 metagenome-assembled genomes (MAGs) revealed two distinct groups of “*Ca.* Bipolaricaulota” MAGs based on the differences in carbon metabolism: one group encoding the bacterial Wood-Ljungdahl pathway (WLP) for H_2_ dependent CO_2_ fixation is selected for at lower salinities, and a second heterotrophic clade that lacks the WLP that was selected for under hypersaline conditions in the geothermal brine sediment. In conclusion, our results highlight that the biogeography of “*Ca.* Bipolaricaulota” taxa is strongly correlated with salinity in hydrothermal ecosystems, which coincides with key differences in carbon acquisition strategies. The exceptionally high relative abundance of apparently heterotrophic representatives of this novel candidate Phylum in geothermal brine sediment observed here may help to guide future enrichment experiments to obtain representatives in pure culture.

## Introduction

All of the Earth’s ecosystems are built on the fixation of carbon dioxide into organic carbon by metabolic carbon fixation pathways. Out of seven carbon fixation pathways known to date (Fuchs, 2011, Sánchez-Andrea et al., 2020), the Wood-Ljungdahl pathway (WLP) is found in a wide range of anaerobic bacterial and archaeal lineages. This pathway has been proposed as one of the ancient metabolic carbon fixation pathways where the last common ancestor of all organisms (LUCA) might have been used to fix carbon in anaerobic hydrothermal settings in the early Earth (Fuchs, 2011).

“*Candidatus* Bipolaricaulota” (Hao et al., 2018), formerly known as OP1 (Hugenholtz et al., 1998) and “*Candidatus* Acetothermum autotrophicum” (Takami et al., 2012), are deeply branching in the bacteria domain with members capable of fixing carbon autotrophically *via* an ancient WLP pathway (Takami et al., 2012). The phylogenetic analysis of the carbon monoxide dehydrogenase enzyme of “*Ca.* Bipolaricaulota,” a key feature of the CO_2_ fixation in the WLP, placed this as one of the most ancient carbon-fixing organisms that have been detected to date (Takami et al., 2012). A recent metagenomic study investigating “*Ca.* Bipolaricaulota” in a serpentinization-driven ecosystem also concluded that this phylum is among the earliest evolving bacterial lineages (Colman et al., 2022). These suggested that members of the “*Ca.* Bipolaricaulota” retain ancestral traits of the WLP that evolved on the early Earth, and the WLP is a metabolism that has been predicted for the LUCA (Martin et al., 2008; Fuchs, 2011). “*Ca.* Bipolaricaulota” have been found in hot springs (Hugenholtz et al., 1998; Tobler and Benning, 2011), deep-sea hydrothermal environments (Teske et al., 2002; Stott et al., 2008), subsurface thermophilic mat communities (Takami et al., 2012), oil fields (Hu et al., 2016), the sediment of hypersaline lagoons (Fernandez et al., 2016), serpentinized subsurface fluids (Colman et al., 2022), and anaerobic bioreactors (Hao et al., 2018). Based on its phylogenomic divergence, it has been proposed as a novel candidate phylum (Hao et al., 2018).

Recently, a complete metagenome-assembled genome (MAG) of the phylum “*Ca.* Bipolaricaulis” anaerobic sp. Ran-1 was obtained from an anaerobic sludge digestor, and this MAG showed a potential chemoheterotrophic mode of lifestyle where members of this bacterium derive energy primarily *via* heterotrophic fermentation (Hao et al., 2018). Moreover, the first photographs of these cells revealed an unusual morphology that exhibited bipolar prosthecae (Hao et al., 2018). The WLP was reported to be ubiquitous in the candidate phylum (by analyzing 14 MAGs), and used in four different catabolic capabilities, namely, (1) homoacetogenic fermentation, (2) autotrophy, (3) syntrophic oxidation of acetate, and (4) respiratory capacity while using oxygen or nitrate as a terminal electron acceptor (Youssef et al., 2019). As such, metabolically versatile memberes of the candidate phylum Bipolaricaulota (Acetothermia, OP1) likely uses the WLP in homoacetogenic fermentation (Youssef et al., 2019).

During 2019–2021, we performed a survey of microbial diversity in geothermal environments on the Biga Peninsula, Turkey, a geothermal field spanning more than 9,000 km^2^. We examined the genomic potential within microbial communities from 11 different geothermal environments in the Biga Peninsula and around the vicinity of Edremit from diverse geothermal conditions including brine pools and subsurface (60–1,350 m below surface) hydrothermal aquifers. This survey revealed several environments where “*Ca.* Bipolaricaulota” reached notably high levels of relative abundance in the microbial communities (>90%). We proceeded to investigate how the diverse geothermal and physicochemical conditions of the Biga Peninsula influence the distribution of “*Ca.* Bipolaricaulota”. By using shotgun metagenomic techniques and bioinformatic tools, four MAGs associated with “*Ca.* Bipolaricaulota” were assembled and their carbon metabolism and biogeographic distribution across the geothermal environments was assessed. Our analyses show how the carbon metabolism of this novel phylum is distributed across key geothermal environments.

## Materials and methods

### Field sampling

**FIGURE 1:**
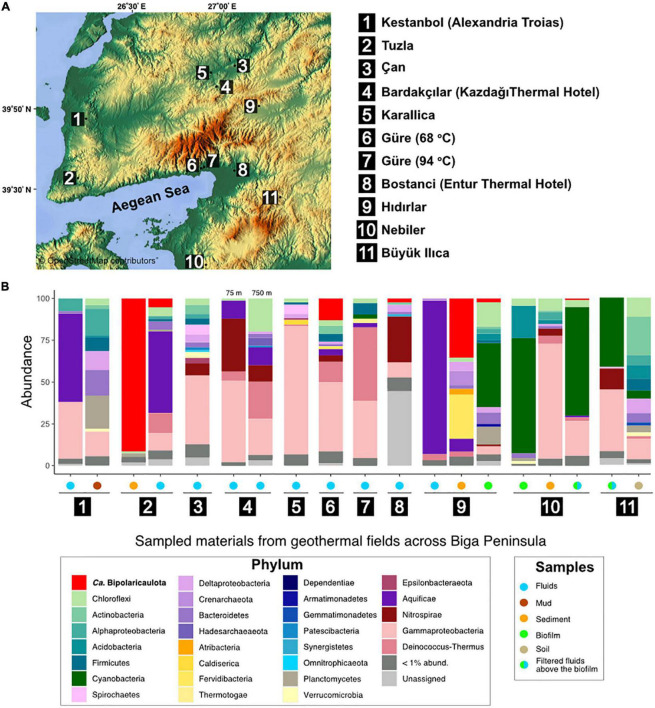
Study sites and microbial community structure. **(A)** Map showing the location of the 11 geothermal sampling sites in Biga Peninsula, Turkey. The legend shows the names of the samples corresponding to the numbers on the map. **(B)** Microbial community structure of collected samples (filtered fluids, sediments, mud, and biofilm samples) from the studied geothermal represented as relative abundance of 16S rRNA gene sequences (y axis). These bars are only displayed for those samples where a phylum/class reached relative abundance in the 16S rRNA gene dataset >1% in any of the samples. Legends show corresponding groups (left) and type of samples (right). The map used in this figure was created by Maps-For-Free which is credited in the image (https://maps-for-free.com/#close).

Sediment and fluid samples were collected in the summertime between 2019 and 2021 from geothermal springs and boreholes in the Biga Peninsula and in the vicinity of Edremit Town (Balikesir, Turkey) (Figure 1 and Supplementary Table 1). Hydrothermal sediments (Tuzla, Hidirlar, Büyükılıca, and Nebiler) were collected directly from the hot spring ponds using falcon tubes or with a shovel. Hot spring fluids (10–20 L) were collected from geothermal pools directly from the spring and immediately filtered (0.2 μm; Millipore Express, Merck, Darmstadt, Germany) from three sites, namely, Tuzla, Hidirlar, and Nebiler, using a peristaltic pump (Masterflex E/S 07571-05, Cole Parmer, USA). Several geothermal fluids were sampled from existing boreholes where deeply sourced hydrothermal aquifer water is constantly pumped to the surface. These fluids were collected either from a water collector (Güre 250 m-deep borehole) or directly from the pipe opening (Güre 1,390 m-deep borehole, Bardakcilar boreholes, Can, and Entur). Additional information on the fluid chemistry boreholes is found in Supplementary Table 1.

### Physicochemical analyses

Electrical conductivity (Ec) (WTW, Cond 3110 Set 1, Weilheim, Germany), temperature, salinity (WTW, Cond 3110 Set 1, Weilheim, Germany), pH (WTW, pH 3110 Set 2, Weilheim, Germany), dissolved oxygen (WTW Oxi 3310, Weilheim, Germany), and total dissolved solids (TDS) were measured in the field using handheld probes. Fluids were filtered through 0.2-μm hydrophilic polyethersulfone (PES) filters (Millipore Express, Merck, Darmstadt, Germany) placed within in-line filter holders. Filters were stored in 15 mL sterile falcon tubes and immediately placed on dry ice, shipped back to Germany, and stored at −80°C until DNA extractions at the University of Munich (LMU Munich). Geochemical analysis of the fluids was performed as described previously (Einsiedl et al., 2020; Supplementary Table 1). Briefly, samples for laboratory-based measurements of major anion and cation concentrations and water isotopes (δ^2^H and δ^18^O) were collected in 1.5 mL tubes after filtering with 0.2 μm PES filters (Millipore Express, Merck, Darmstadt, Germany). All samples were stored on dry ice. Laboratory measurements were done in the hydrogeology department of the Technical University of Munich (TUM) and the General Directorate of Mineral Research and Exploration of Turkey (MTA) (Supplementary Table 1).

### DNA extraction

DNA was extracted as described previously for filters (Orsi et al., 2015) and sediments (Coskun et al., 2018). DNA from the sediments was performed exactly as described previously (Coskun et al., 2018). For the filters, some modifications to the original DNA extraction protocol (Orsi et al., 2015) were made. For the filters, the silica bead contents from four 2 mL Lysing Matrix E tubes (MP Biomedicals, Solon, OH, USA) was poured into a 15-ml falcon tube containing the filter 4 ml of a sterile-filtered sucrose ethylene-diaminetetraacetic lysis buffer (0.75 M sucrose, 0.05 M Tris-Base, 0.02 M ethylenediaminetetraacetic, 0.4 M NaCl, 4 ml 10% sodium dodecyl sulfate, and pH 9.0) was added to the tubes and then bead beating was performed for 40 s using a Fast-Prep 5G homogenizer (MP Biomedicals, OH, USA) at a speed of 6 m/s. Samples were subsequently heated for 2 min at 99°C. After heating, 25 ml of 20 mg/ml proteinase K was added, and tubes were incubated at 55°C overnight with constant gentle rolling in a Bambino oven. DNA was extracted and purified from the lysate using the DNeasy Blood and Tissue Kit (Qiagen). Extracted DNA was quantified fluorometrically using a Qubit 3.0 Fluorometer (Invitrogen, Eugene, OR, USA).

### PCR of 16S rRNA genes and bioinformatic analysis

Universal primers targeting the V4 hypervariable region of 16S ribosomal RNA (rRNA) genes were used to PCR amplify the gene fragments from DNA extracts from the environmental samples. We used a version of the 515F primer with a single-base change (in bold) to increase the coverage of certain taxonomic groups including the archaea (515F-Y, 5′-GTG**Y**CAGCMGCCGCGGTAA; Parada et al., 2016). PCR reactions were carried out as described previously (Coskun et al., 2019). The 16S rRNA genes were subjected to dual-indexed barcoded sequencing of 16S rRNA gene amplicons on the Illumina MiniSeq as described previously (Pichler et al., 2018).

The MiniSeq reads (Pichler et al., 2018) were quality trimmed and assembled using USEARCH version 11.0.667 with the default parameters (Edgar, 2010). Reads were then *de novo* clustered at 97% identity using UPARSE; operational taxonomic units (OTUs) represented by a single sequence were discarded (Edgar, 2013). Taxonomic assignments were generated by QIIME 1.9.1 (Caporaso et al., 2010) using the implemented BLAST method against the SILVA rRNA gene database release 132 (Quast et al., 2013). The genera *Pseudomonas*, *Ralstonia*, *Variovorax*, or *Streptococcus* were also removed as these are common contaminants of molecular reagent kits (Salter et al., 2014) and we typically find these genera in DNA extraction blanks from our lab (Coskun et al., 2018; Pichler et al., 2018). Statistical analyses and plots were performed using R. Studio version 3.3.0 (RStudio Team, 2015). The 16S rRNA gene sequence data is stored in the NCBI Short Read Archive under BioProject ID PRJNA888248.

### Metagenomic analysis

Samples selected for metagenomic shotgun sequencing (see Supplementary Figure 1) were prepared into metagenomic libraries using the Nextera XT DNA Library Prep Kit (Illumina) by following the manufacturer instructions with some modifications. The starting concentration of genomic DNA could not be set to 0.2 ng as suggested by the manufacturer due to low DNA content in some samples. Instead, the PCR program in the amplification step of the tagmented DNA was modified from 12 to 15 cycles to increase the resulting DNA yield from <1 nM concentration to the values between 5 and 10 nM as previously done (Coskun et al., 2021). Quality control and quantification of the metagenomic libraries were done on an Agilent 2100 Bioanalyzer System using high-sensitivity DNA reagents and DNA chips (Agilent Genomics). Metagenomic libraries were diluted to 1 nM and pooled together to be sequenced on the Illumina MiniSeq platform. SqueezeMeta (Tamames and Puente-Sánchez, 2018) and the Anvi’o snakemake workflow (Köster and Rahmann, 2012; Eren et al., 2015) were used for downstream analysis in co-assembly mode with default settings. In short, the SqueezeMeta workflow deployed Trimmomatic for adapter removing, trimming, and quality filtering by setting the parameters: leading = 8, trailing = 8, sliding window = 10:15, and minimum length = 30 (Bolger et al., 2014). Contigs were assembled using a Megahit assembler with a minimum length of 200 nucleotides (Li et al., 2015). Open reading frames (genes and rRNAs; ORFs) were called using Prodigal (Hyatt et al., 2010); rRNAs genes were determined by Barrnap^1^. The Diamond software (Buchfink et al., 2015) was used to search for gene homologies in the databases GenBank nr for taxonomic assignment, eggNOG v4.5 (Huerta-Cepas et al., 2016), and KEGG (Kanehisa, 2000). The cutoff values for assigning hits to specific taxa were performed at an e value of 1 × e^–3^ and a minimum amino acid similarity of 40 for taxa and 30 for functional assignment, which was the default settings of SqueezeMeta. Bowtie2 (Langmead and Salzberg, 2012) was used to map the read onto contigs and genes. Anvi’o snakemake workflow (anvi’o v7) was used to bin and refine the MAGs. MaxBin (Wu et al., 2016), CONCOCT (Alneberg et al., 2014), and Metabat2 (Kang et al., 2019) were used for the binning, and the DAS Tool (Sieber et al., 2018) was used to choose the best bin for each population. The completeness and contamination of the bins were checked in anvi’o v7 based on the “Bacteria_71” single-copy gene database embedded in anvi’o v7. The metagenomic sequence data is stored at https://doi.org/10.6084/m9.figshare.21286227.v1 and entered in the NCBI Short Read Archive under BioProject ID PRJNA888248.

For the pangenomic analysis, the MAGs to compare against our study were downloaded from public databases deposited as a part of research efforts (Takami et al., 2012; Jungbluth et al., 2017; Parks et al., 2017; Hao et al., 2018; Kadnikov et al., 2019; Smith et al., 2019; Youssef et al., 2019; Liu et al., 2020; Reysenbach et al., 2020; Zhou et al., 2020; Bhattarai et al., 2021; Schneider et al., 2021; Wang et al., 2021; Brazelton et al., 2022; Speth et al., 2022). Supplementary Table 2 provides information on the 23 origins of all the genomes examined in this study.

### Phylogenomics of “*Ca.* Bipolaricaulota”

A total of 69 different sets of ribosomal genes (in total) were determined by anvi’o (Eren et al., 2015) using the “Bacteria_71” ribosomal protein dataset in anvio’s “anvi-get-sequences-for-hmm-hits” command (Eren et al., 2015). These ribosomal proteins were aligned using muscle (Edgar, 2004), and the phylogenetic tree was constructed using FastTree Version 2.1.10 (Price et al., 2010) using BLOSUM45, which is embedded in anvi’o (Eren et al., 2015).

### Statistical analysis

**FIGURE 2:**
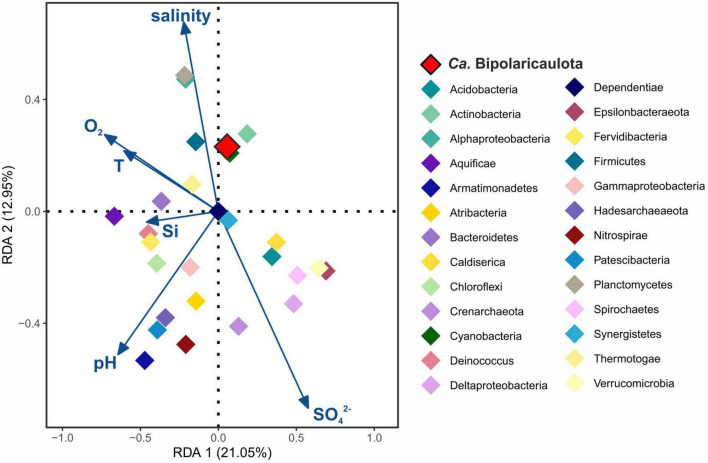
Redundancy analysis (RDA) ordination plot for Biga Peninsula Phylum operational taxonomic units (OTUs) distribution constrained by six environmental variables. Environmental parameters (blue arrows) included: oxygen concentration (O_2_), temperature (T), pH, salinity, silica (Si), and sulfate (SO_4_^2–^) concentration. Color codes of the different phylum are the same as in Figure 1, being “*Ca.* Bipolaricaulota” highlighted in red.

For the statistical assessment of the influence of the environmental parameters on “*Ca.* Bipolaricaulota” and the rest of the identified phyla in the fluids, we applied a redundancy analysis (RDA) (Figure 2). RDA is a multiple linear regression between the explanatory variables (environmental parameters) and the response variables (OTUs abundance per different phylum), followed by a principal component analysis (PCA). Sediment, biofilm, mud, and soil samples were discarded from the analysis as physicochemical parameters were only measured from the fluids. Previous to the RDA plot, Pearson correlations (*r* > 0.8, *P* < 0.05) were applied to all physicochemical parameters (Supplementary Table 1) and only those parameters that did not correlate among themselves (O_2_, T, pH, salinity, [ < *cps*:*it* > *Si* < /*cps*:*it* > ] and [SO_4_^2–^]) were included in the further regression. A total of 177 OTUs grouped into 27 different phyla were included in the RDA plot, including those OTUs representing >1% of the relative abundance in any sampling site (Figure 2 and Supplementary Table 1). Statistical analyses were performed using the R software package using the *corrplot* package (Wei et al., 2013) and the *vegan* package (Oksanen et al., 2013), and plots were displayed using the *ggplot2* package (Wickham, 2016).

## Results

### Fluid geochemistry and the origin of thermal waters in Biga Peninsula

**Figure 3:**
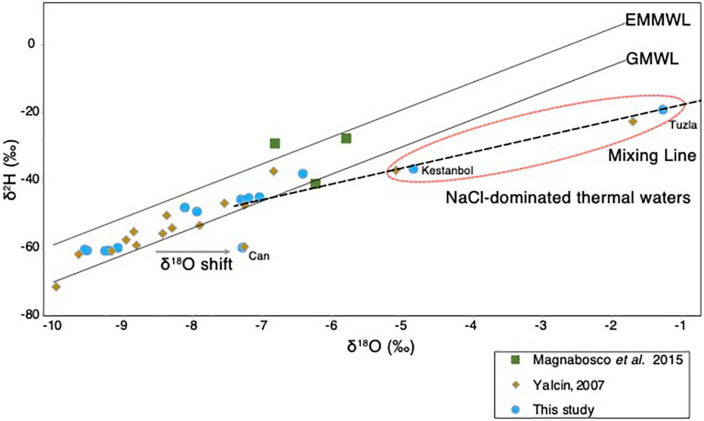
Isotopic composition of the geothermal waters. The δ^2^H (‰) and δ^18^O (‰) plot of thermal waters obtained in the study (blue), and other studies (Yalcin, 2007; Magnabosco et al., 2016). The GMWL and EMMWL stand for Global Meteoric Water Line (Craig, 1961) and East Mediterranean Water Line (Gat and Carmi, 1970). The hypersaline brine waters at the Tuzla site (Figure 1) that do not fall on the EMMWL or GMWL are highlighted in red circle.

The origin of the different geothermal fluids sampled from different sites in the Biga Peninsula (Figure 1A) was assessed using stable isotopic composition, δ^2^H and δ^18^O, of the geothermal fluids (Figure 3). The results showed that the isotopic composition of most samples fell between the global meteoric water line (GMWL; Craig, 1961) and East Mediterranean meteoric water line (EMMWL; Gat and Carmi, 1970). The exceptions to this trend were geothermal fluids sampled from Kestanbol, Tuzla, and Can (Figure 3), as their δ^2^H vs. δ^18^O values did not plot between the EMMWL and GMWL (Figure 3). The geothermal fluids sampled from Tuzla and Kestanbol were likely affected by higher salinities, as they had higher conductivity values and were enriched with high NaCl concentrations, whereas the other sites were dominated by NaSO_4_-enriched waters (Supplementary Table 1). The Ec value of the Kestanbol geothermal field was between the definition of high saline waters (∼15,000 to ∼55,000 μS/cm according to USGS standards), which equals a salinity of 20 g/kg. On the contrary, the measured conductivity of the geothermal fluid at Tuzla (80,000 μS/cm) showed that the sampled fluids were brine, as this Ec value corresponds to a salinity of 55 g/kg, which is much higher than the Mediterranean seawater (38 g/kg) (Nessim et al., 2015).

### 16S rRNA gene profile of hydrothermal ecosystems in the studied area

Across 11 geothermal sites from the Biga Peninsula and the vicinity of Edremit 29 phyla were detected with relative abundance higher than 1% in the 16S rRNA gene datasets (Figure 1B). The overall microbial community from the studied geothermal sites, which display a broad spectrum of different geochemical conditions (Supplementary Table 1), was dominated by OTUs affiliated with known thermophilic groups within the Gammaproteobacteria, Deinococcus-Thermus, Chloroflexi, Aquificae, “*Ca.* Bipolaricaulota”, Planctomycetes, Cyanobacteria, and Acidobacteria (Figure 1B).

OTUs affiliated with Gammaproteobacteria (particularly uncultured members of the family Hydrogenophilaceae) were found to be relatively dominant in filtered hydrothermal fluids from Can (40.9%), Güre (41.1%), Karailica (76%), Bardakcilar (21.6 and 48.7% in 750 and 75 m-deep boreholes, respectively), Kestanbol (34%), and a sediment sample from Nebiler (68.7%). OTUs affiliated with the Aquificae dominated the microbial community in the filtered fluids from Hidirlar (91.8%; mainly *Hydrogenobacter* sp.), Kestanbol (52.8%, *Hydrogenivirga* sp.), and Tuzla (48.5%, *Hydrogenivirga* sp.), and were well represented in Bardakcilar geothermal fluids from 75 and 750 m-deep boreholes with 10% relative abundance. Bacterial OTUs affiliated with the Deinococcus-Thermus phylum were also relatively abundant in the filtered fluids from the deepest sampled well from this study (1,390 m with a temperature of 94°C) in Güre town (43.9%), 750-m-deep borehole in Bardakcilar geothermal field (22.2%), and were relatively abundant in the fluids of Tuzla village (11.8%) and Güre 260 m-deep borehole (12.1%). On the contrary, biofilms and filters taken above the biofilms from hot springs of Buyukilica, Hidirlar, and Nebiler geothermal fields were predominated by OTUs associated with Cyanobacteria with relative abundances ranging from 38.4 to 68.9%.

The relative abundance of the total 16S rRNA gene sequence reads assigned to “*Ca.* Bipolaricaulota” varied substantially between the different sites sampled and ranged from <1 to 91.5% in terms of their fractional abundance of total 16S rRNA gene sequences (Figure 1). The highest abundance of “*Ca.* Bipolaricaulota” (5 abundant OTUs; 91.5% of the total reads) was found in the sediments of a 68.1°C brine pool (Tuzla) (Figure 1). Moreover, the “*Ca.* Bipolaricaulota” in sediment samples from the 78°C Hidirlar geothermal field had a 35.5% relative abundance. OTUs affiliated with “*Ca.* Bipolaricaulota” were also detected in all of the sampled subsurface geothermal wells, extending down to a depth of 1,300 m below the Earth’s surface at the Güre borehole (Figure 1) and were relatively abundant specifically in a 260 m-deep borehole in the Güre region with 12.9% relative abundance.

### “*Ca.* Bipolaricaulota” distribution in geothermal environments correlated with physicochemical parameters

RDA analysis showed that the major microbial groups detected at the Biga Peninsula were differently affected by the environmental conditions of the fluids, as they were clustered in different ordination spaces that positively or negatively related to different geochemical parameters (salinity, pH, O_2_, Si, SO_4_^2–^, and temperature) in the RDA (Figure 2). The first RDA axis explained 21.05% of the OTUs variability and the second RDA axis explained 12.95%. The distribution of “*Ca.* Bipolaricaulota” OTUs in the various geothermal environments sampled (Figure 1) was positively related to salinity and negatively related to pH and sulfate concentration (Figure 2). Out of all five key physicochemical parameters tested (salinity, pH, O_2_, Si, SO_4_^2–^, and temperature), salinity had the strongest relationship with the biogeographic distribution of “*Ca.* Bipolaricaulota” taxa (Figure 2).

### Microbial composition within metagenomes and metagenome-assembled genomes

In line with the 16S rRNA gene-based microbial community structure (Figure 1), taxonomical annotations of open reading frames (ORFs) from the samples showed that microbial communities were dominated by the Aquificae, Proteobacteria, Nitrospirae, Deinococcus-Thermus, Bacteroidetes, and Crenarchaeota at different proportions (Supplementary Figure 1). On the contrary, the differences in relative abundances of microbial communities between 16S rRNA profiles and ORFs showed unexpected discrepancies, especially in the samples where members of “*Ca.* Bipolaricaulota” were detected (Figure 1 and Supplementary Figure 1). For example, sediment samples from Tuzla brine exhibited a 4% relative abundance of ORFs annotated as “*Ca.* Bipolaricaulota” (Supplementary Figure 1), whereas the 16S rRNA gene sequences from this sample was dominated by this candidate phylum (Figure 1). It should be noted that based on the 60% similarity threshold of ORFs in Squeezemeta (Tamames and Puente-Sánchez, 2018), some ORFs could not be taxonomically assigned whose relative abundances reached around 50% in Tuzla brine (Supplementary Figure 1). 137 MAGs were assembled from the samples taken from the Biga Peninsula and the vicinity of Edremit town (Supplementary Tables 3, 4), which were used to investigate the metabolic potential of “*Ca.* Bipolaricaulota” in more detail.

### MAGs affiliated with “*Ca.* Bipolaricaulota”

**FIGURE 4:**
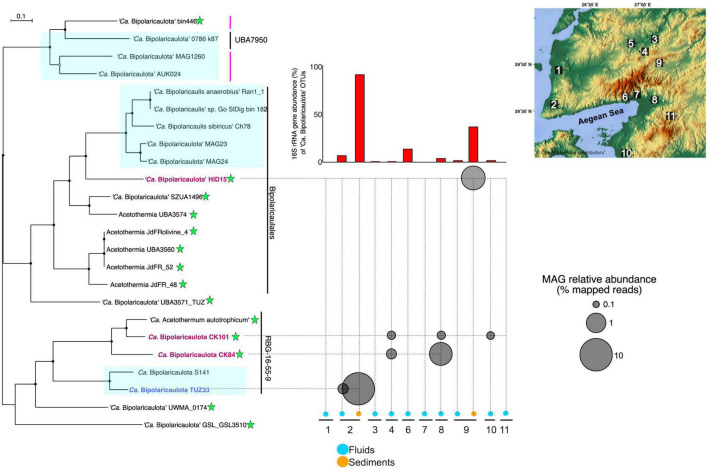
The phylogenetic tree is an analysis of concatenated 69 ribosomal proteins extracted from 23 metagenome assembled genomes affiliated to ‘*Ca.* Bipolaricaulota’. Black circles at nodes represent bootstrap support of 90%, gray circles represent bootstrap support from 70 to 90%, and white circles represent bootstrap support from 70 to 50%. Blue boxes represent the MAGs with no Wood-Ljungdahl pathway (WLP) encoded. The green stars show the MAGs containing the WLP. Bold and colored fonts represent the MAGs assembled from the geothermal sites in this study (dark blue and red). The bubble plot shows the relative abundance of the “*Ca.* Bipolaricaulota” MAGs assembled in this study corresponding to their position in the phylogenetic tree. The histograms above the bubble plot show the relative abundance of ‘*Ca.* Bipolaricaulota’ from 16S rRNA gene datasets for each sampling location. The map represents the sampling locations, which are also shown in Figure 1 and Supplementary Figure 1.

Out of a total of 137 MAGs, four of them were taxonomically assigned to “*Ca.* Bipolaricaulota”, with completeness ranging from 91.5 to 83% (0% redundancy) and redundancy varying from 0 to 4% based on the presence of single copy genes (see the Section “Materials and methods”) (Supplementary Table 2). Read mapping against these MAGs revealed that they had a relatively high abundance in the metagenomes, reaching up to 10% of binned total reads in the Tuzla geothermal brine sample (Figure 4). This high relative abundance of the “*Ca.* Bipolaricaulota” TUZ33 MAG in the Tuzla brine sediments coincided with a high relative abundance of “*Ca.* Bipolaricaulota” 16S rRNA gene OTUs in this same sample that reached >90% relative abundance (Figure 1). In contrast, the other three “*Ca.* Bipolaricaulota” MAGs (CK84, CK101, and HID15) were largely restricted to geothermal fluids with much lower salinities (measured as Ec) (Figure 4 and Supplementary Table 4). Of these MAGs, the HID15 had the highest relative abundance at the Hidirlar geothermal sediments, whereas MAGs CK84 and CK101 had their highest relative abundances in the subsurface borehole Entur (300 m) (Figure 4 and Supplementary Table 4).

A phylogenomic analysis of a concatenated single-copy gene alignment revealed the phylogenetic relation of the MAGs sampled in our study to existing MAGs from other studies (Figure 4). Two MAGs (CK101 and CK84) from the less-saline Turkish geothermal sites had the closest phylogenetic affiliation to the deeply branching *Candidatus* ‘Acetothermum autotrophicum’ (Takami et al., 2012). MAG HID15 with the highest relative abundance in the geothermal sediments from the Hidirlar site (Figure 1) was affiliated with Order Bipolaricaulales and branched basal to a larger clade, including the Ran_1 MAG that was recovered from anaerobic sludge (Hao et al., 2018). The fourth MAG (TUZ33) that was highly abundant in the geothermal brine pool from Tuzla (Figure 1) branched basal to the *Candidatus* ‘Acetothermum autotrophicum’ clade and was clustered within Order RBG-16-55-9 (Figure 4).

### WLP and energy metabolism of “*Ca.* Bipolaricaulota” genomes

**FIGURE 5:**
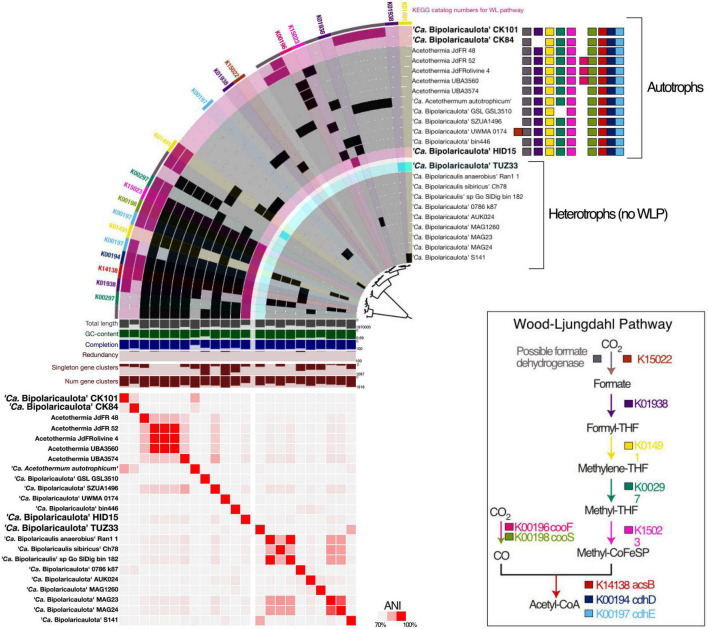
Comparative genomic analysis of gene clusters encoding Wood-Ljungdahl pathway (WLP) **(top row)**, and those lacking the WLP **(bottom row)**. The metagenome-assembled genomes (MAGs) recovered in this study are shown with bold letters and bigger font size; the MAGs taken from the public databases are displayed in black with relatively smaller font size. The heatmap shows average nucleotide identity between MAGs across the entire core genome. Values were calculated in the anvio v7 using pyANI (Pritchard et al., 2016). The colored squares represent the enzymes catalyzing the WLP.

We compared the “*Ca.* Bipolaricaulota” MAGs reconstructed from the Turkish geothermal sites (Figure 5 and Supplementary Figure 2) to most of the publicly available mid/high-quality “*Ca.* Bipolaricaulota” MAGs using pangenomic analysis in order to better understand the carbon metabolism of this phylum (Supplementary Tables 2, 5). The pangenome analysis uncovered that pairwise average nucleotide identities were spanning from nearly 65 to 99.9% in the “*Ca.* Bipolaricaulota” phylum (Figure 5 and Supplementary Table 2). The MAG from the Turkish geothermal fluids with the highest average nucleotide identity to the deeply branching *Candidatus* ‘Acetothermum autotrophicum’ (Takami et al., 2012) was found to be CK101 (Figure 5). The pangenomic analysis shows that the core genome within “*Ca.* Bipolaricaulota” is relatively small compared to the flexible genome content of the individual MAGs (Supplementary Figure 2). The majority of the “*Ca.* Bipolaricaulota” pangenome is dominated by singleton genes (those genes detected in only one MAG and not any other MAGs) (Supplementary Figure 2). Pathways for fermentation were prevalent in the “*Ca.* Bipolaricaulota” MAGs (Supplementary Figure 2).

The pangenomic analysis identified two groups of “*Ca.* Bipolaricaulota” MAGs: one that contained the WLP pathway for H_2_-dependent CO_2_ fixation (Schuchmann and Müller, 2016) and another group of “*Ca.* Bipolaricaulota” MAGs that did not contain this pathway (Figure 5; Supplementary Figure 2 and Supplementary Table 5). The concatenated single-copy gene alignment revealed that the presence or absence of the WLP was restricted to particular clades and branches, and did not correlate with a monophyletic pattern (Figure 5). From our Turkish geothermal sites, three of the MAGs (CK84, CK101, and HID15) contained the WLP pathway (Figure 5 and Supplementary Figure 2), whereas the fourth MAG (TUZ33) did not encode it (Figure 5 and Supplementary Figure 2). Pyruvate:ferredoxin oxidoreductases were found in 17 MAGs, 12 of which had complete/incomplete genes encoding WLP components (CK101, CK84, jdFR48, jdFR52, Olivine4, UBA3560, UBA3574, *Candidatus* ‘Acetothermum autotrophicum’, GSL_GSL3510, SZUA1496, UMWA_0174, and HID15) (Supplementary Table 5). A total of 12 MAGs encoded membrane-bound hydrogenases (alpha, beta, and mbhJ were observed) [EC:1.12.7.2]. In addition, 10 WLP-encoding MAGs had subunits of heterodisulfide reductase, which is involved in electron bifurcation (Buckel and Thauer, 2018; Supplementary Table 5). Moreover, all MAGs had the capacity for multiple fermentation pathways (Supplementary Figure 2).

## Discussion

Despite featuring more than 2,000 geothermal sources within 460 geothermal fields (Mertoglu et al., 2020), the geothermal environments of Turkey are historically undersampled for microbial communities. There are few studies from geothermal springs and aquifers in Turkey that have mainly applied cultivation-based techniques that have isolated thermophilic organisms (Belduz et al., 2003; Çelikoğlu and Cankılıç, 2016; Guven et al., 2018). However, how the microbial diversity and metabolism relates to the diverse physicochemical conditions of the numerous geothermal environments is still poorly understood.

### The origin of the thermal waters in the Biga Peninsula

The origin of geothermal waters in the Biga Peninsula was determined using the stable isotopic composition, δ^2^H and δ^18^O, of the fluids (Figure 3). The results showed that the isotopic composition of most samples, together with deep water from a South African gold mine (Magnabosco et al., 2016), fell between the global meteoric water line (GMWL; Craig, 1961) and the East Mediterranean meteoric water line (EMMWL; Gat and Carmi, 1970), suggesting that the subsurface hydrothermal water had been replenished by modern meteoric water (Figure 3). An exception to this trend was found in the samples from Tuzla, Kestanbol, and Can, which had higher salinities and did not plot on the EMMWL and GMWL (Figure 3). These results were also in line with the findings published by Yalcin (2007). Samples taken from Kestanbol and Tuzla contained high amounts of sodium and chloride, hence they have been classified as NaCl-dominated waters (Yalcin, 2007). The stable isotopic composition of the thermal waters in these areas, δ^2^H and δ^18^O, fell onto a mixing line between meteoric waters and deep-seated hot brine waters whose origin is not known, which is consistent with the previous study (Yalcin, 2007). It has been proposed that the origin of water could be the fossil water trapped in Miocene sediments (Balderer, 1997), whereas another study stressed that hypersalinity in these waters could result from the dissolution of salt deposits (Vengosh et al., 2002). In our study, we identified a δ^18^O-shift in Can thermal water, most likely explained by CO_2_ input from a geological source, which was also suggested by a previous study that investigated thermal waters in the Biga Peninsula (Yalcin, 2007).

### Carbon metabolism of “*Ca.* Bipolaricaulota”

Our pangenome analysis indicates that the WLP is restricted to a subset of clades within the ‘*Ca.* Bipolaricaulota’ (Figures 4, 5). The WLP can function in reductive (CO_2_-reduction to acetyl-CoA) and oxidative (acetate utilization) directions depending on the metabolic demands of a microorganism. The reductive WLP is used in the reductive direction for energy conversion and autotrophic carbon fixation in acetogens and methanogens (Ragsdale and Pierce, 2008). The WLP may be involved in homoacetogenic fermentation during the anaerobic oxidation of sugars and amino acids (Schuchmann and Müller, 2016), coupled with autotrophy to fix CO_2_ into biomass (Fuchs, 2011). However, it is possible that the WLP is reversible and in the reverse direction, it would potentially function as oxidation of acetate, producing CO_2_ and H_2_ as end products (Müller et al., 2013). In oxidative WLP, acetate is oxidized to form CO_2_ and H_2_, which is performed by syntrophic acetate-oxidizing bacteria (SAO) when growing with hydrogenotrophic methanogens. Thus, the WLP provides exceptional metabolic flexibility to the organisms if they can use it in both directions.

H_2_ production during fermentation of organic matter by “*Ca.* Bipolaricaulota” was indicated for a strain inhabiting anaerobic sludge, where it might support syntrophic interactions with H_2_ utilizing methanogenic archaea and/or sulfate-reducing bacteria (Hao et al., 2018).

The WLP leads from two molecules of CO_2_ that are reduced *via* H_2_ making acetyl-CoA, which can contribute to biomass synthesis, ATP production via substrate-level-phosphorylation, and also provides reducing power to drive a proton motive force at the membrane for ATP synthesis (Schuchmann and Müller, 2016). It is a key metabolism for many homoacetogenic bacteria and helps to increase the efficiency of ATP production via fermentation under energy-limited conditions, such as those in anoxic environments lacking high-energy terminal electron acceptors with extremely low redox potential (Schuchmann and Müller, 2016). In our pangenome analysis of the WLP in “*Ca.* Bipolaricaulota”, we identified many key WLP genes, including *acsB* (K14138), *cdhD* (K00194), and *cdhE* (K00197). However, some necessary genes for the WLP were missing in most MAGs (Figure 5; Supplementary Figure 2 and Supplementary Table 5), whereas the complete WLP was detected in the “*Ca.* Bipolaricaulota” MAGs JdFR 48, JdFR 52, and *Acetothermium autotrophicum.* This is in accordance with prior results (Youssef et al., 2019). Our phylogenomic analysis shows that the WLP is specific to particular sub-groups of “*Ca.* Bipolaricaulota” but is not phylum-wide distributed. The WLP containing MAGs in several geothermal environments of the Biga Peninsula can apparently fix carbon dioxide (CK101, CK84, and HID15) (Figure 5), but also have the genomic capacity for fermentation (Supplementary Figure 2). Therefore, their energy metabolism bears a resemblance to homoacetogens that use the WLP to harness CO_2_ as an electron sink to improve ATP production efficiencies under energy-limited conditions (Schuchmann and Müller, 2016).

### Salinity controls the distribution of “*Ca.* Bipolaricaulota” in geothermal fluids

The highest abundance of “*Ca.* Bipolaricaulota” was found in the sediments of a geothermal brine pool at the Tuzla site, and our statistical analysis shows that OTUs affiliated with “*Ca.* Bipolaricaulota” correlated most strongly with salinity compared to five other key physicochemical parameters such as temperature, O_2_, pH, Si, and SO_4_^2–^ (Figure 2). This indicates that salinity is a key driver of the biogeographic distribution for some members of this particular clade. Notably, the “*Ca.* Bipolaricaulota” MAGs that were dominant in this brine setting are lacking the WLP, indicating that they are heterotrophs (Figure 5). In line with our analysis, it has been reported that the Tuzla sample contains nearly twice the amount of salt concentration compared to the same concentrations in seawater as sodium and chloride concentrations, which reach 17 and 68 mg/L, respectively (Baba et al., 2009). These findings are consistent with other studies that found an affinity of this group for brine environments such as in Orca Basin (Nigro et al., 2016), Hephaestus and Kryos basins (Fisher et al., 2021), hypersaline stratified layers of Ursu Lake (Baricz et al., 2021), saline pan sediments in the Kalahari Desert of southern Africa (Genderjahn et al., 2018), anoxic hypersaline layers of Witpan in South Africa, and in surface sediments of salty Siberian soda lakes (Vavourakis et al., 2018). Our findings show that (a) the WLP encoding (carbon-fixing) “*Ca.* Bipolaricaulota” MAGs are enriched at lower salinities (Figures 1, 4, 5) and (b) they were still detected in the brine at a relatively low abundance, suggesting that they are halotolerant.

### Energy metabolism and survival under extreme conditions

Many of the WLP-encoding MAGs in the pangenome analysis encode fermentation pathways that are known to produce formate, H_2_, and acetate (Supplementary Figure 2). These MAGs also encode the pyruvate: ferredoxin oxidoreductase complex (porABCD) that produces acetyl-CoA from pyruvate, thereby generating reduced ferredoxin. The encoded Acetyl-CoA synthases (acs) can then produce ATP (for energy) and acetate (as a fermentation product) during fermentation (Supplementary Table 5). The potentially produced formate and H_2_ from fermentation processes (Supplementary Figure 2), could be utilized by syntrophic partners (Hao et al., 2018). Also, in line with a recent metagenomic study conducted in Samail Ophiolite in Oman, the fermentation capability of “*Ca.* Bipolaricaulota” MAGs has been shown, especially in MAGs associated with Order RBG-16-55-9, where they dominate alkaline fluids (Colman et al., 2022). The combined potential for fermentation in the MAGs, which also encode the WLP, highlights the genomic capacity of some “*Ca.* Bipolaricaulota” to survive *via* mixotrophy (combining H_2_-dependent CO_2_ fixation *via* the WLP with heterotrophy). The mixotrophic “*Ca.* Bipolaricaulota” might exhibit a homoacetogenic fermentation metabolism, which is a common feature of many anaerobic heterotrophic bacteria living close to the energy limit of life (Schuchmann and Müller, 2016). Specifically, CO_2_ acts as an electron sink (*via* the WLP) during the oxidation of organic matter during fermentation in order to provide a reducing potential to drive a proton motive force at the membrane (Schuchmann and Müller, 2016). The encoded heterodisulfide reductases in the MAGs suggest the potential for electron bifurcation to play a role in linking the fermentation and WLP processes (Schuchmann and Müller, 2016).

The extreme energy limitation of the deep subsurface environments sampled here (up to 1,300 m below the surface) presents a challenge for microbial life to survive. It is proposed that a prosthecate provides a competitive advantage for “*Ca.* Bipolaricaulota” to survive under nutrient-limited conditions by increasing the surface area for nutrient uptake (Hao et al., 2018). This would be important for energy-limited environments such as the deep subsurface. However, the Ran1 MAG from which the prosthecates were observed (Hao et al., 2018) shares less than 70% average nucleotide identity with the MAGs recovered from the Turkish geothermal settings here (Figure 5 and Supplementary Table 2) and is part of a more derived clade within the Bipolaricaulales Order that does not encode WLP (Figure 4). This level of ANI difference likely corresponds to different microbial genera (Konstantinidis and Tiedje, 2005) and indicates the in-group diversity of the proposed novel phylum “*Ca.* Bipolaricaulota” (Hao et al., 2018; Youssef et al., 2019) is relatively large. Moreover, the flexible genomes of the Ran1 MAG and the MAGs from the geothermal aquifers of Turkey show minimal overlap (Figure 5). The large genetic difference and the large differences in flexible genome content raise the possibility that “*Ca.* Bipolaricaulota” in the Turkish geothermal settings has a different phenotype that may not have prosthecate, but additional microscopy is needed to confirm this. Future studies combining metagenomics with microscopy, cultivation, and phylogenomics will help to reveal how phenotypic traits correlate with life histories in the novel candidate phylum “*Ca.* Bipolaricaulota”.

## Data availability statement

The data presented in this study are deposited in https://doi.org/10.6084/m9.figshare.21286227.v1 and in the NCBI Short Read Archive repository, accession number PRJNA888248.

## Author contributions

ÖKC, MB, DO, and HH performed sampling. ÖKC and FE performed lab work. ÖKC, GVG-S, WDO, and FE performed data analysis. ÖKC and WDO designed the study. OKC, GVG-S, and WDO wrote the manuscript. All authors contributed to the article and approved the submitted version.

## Footnotes

1
github.com/tseemann/barrnap
