# Supplementary figures and images for: Carbon metabolism and biogeography of candidate phylum “Candidatus Bipolaricaulota” in geothermal environments of Biga Peninsula, Turkey
Source: Front Microbiol. 2023 Feb 22;14:1063139. doi: 10.3389/fmicb.2023.1063139 (PMC9992828; doi:10.3389/fmicb.2023.1063139)

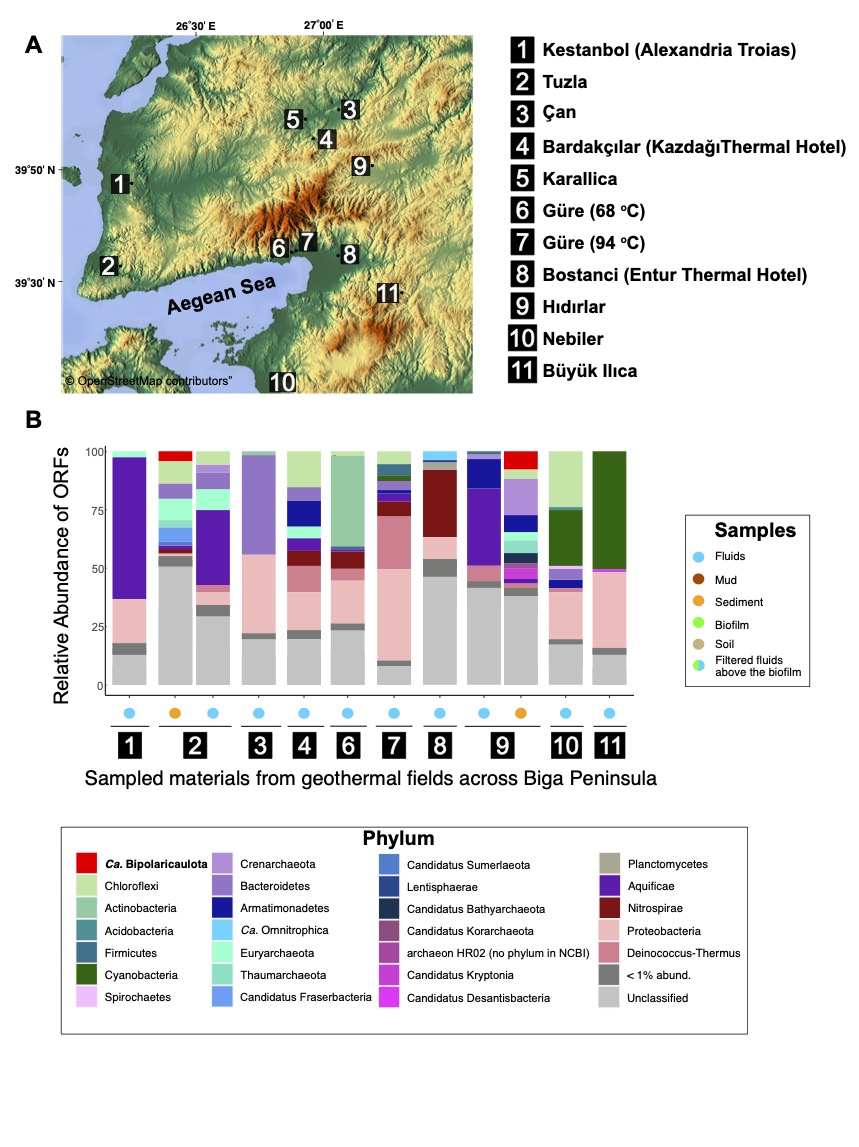

Supplement: Supplementary file 1 [file Image_1.JPEG]

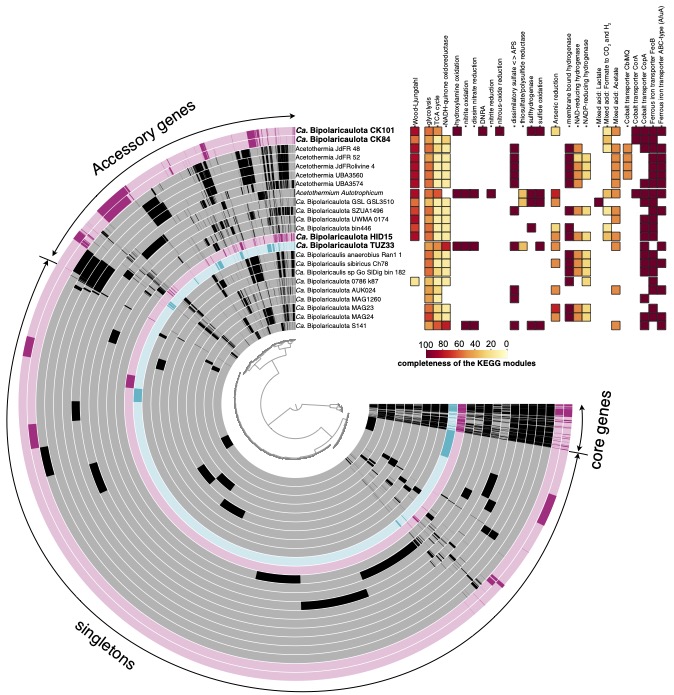

Supplement: Supplementary file 2 [file Image_2.JPEG]
